# Design and Application of an Acetabular Integrative Anatomic Plate: A Retrospective Study of 178 Cases with Complex Acetabular Fractures

**DOI:** 10.1111/os.13817

**Published:** 2023-08-24

**Authors:** Shicai Fan, Qiguang Mai, Tao Li, Hua Wang, Cheng Yang, Hai Huang, Jianwen Liao, Yingze Zhang

**Affiliations:** ^1^ Department of Traumatic Surgery, Center for Orthopaedic Surgery Third Affiliated Hospital of Southern Medical University Guangzhou China; ^2^ Trauma Emergency Center Third Hospital of Hebei Medical University Shijiazhuang China

**Keywords:** Acetabular fracture, Anatomical plate, Integrative, Lateral‐rectus approach

## Abstract

**Objective:**

As conventional plates require repeated pre‐bending during surgery with poor matching, this study aimed to explore the design and application of an acetabular integrative anatomical plate (AIAP) *via* the lateral‐rectus approach (LRA) in fresh complex acetabular fractures for the good reduction and fixation.

**Methods:**

We designed an AIAP based on the anatomical morphology of the Chinese people. From March 2016 to September 2021, 178 patients with fresh complex acetabular fractures treated with an AIAP *via* the LRA were retrospectively analyzed. All patients were treated by the LRA under general anesthesia in a supine position. The fragments were well reduced and fixed by AIAPs. The operation time and intraoperative blood loss were recorded. All patients underwent reexamination of pelvic X‐rays and CT scans and were followed up for over 1 year postoperatively. The reduction quality of fracture was evaluated according to the Matta criteria. The postoperative functional recovery was evaluated by modified Merle d'Aubigne‐Postel scoring system. Statistics were analyzed by SPSS 25.0 (SPSS Inc., Chicago, IL, USA).

**Results:**

All 178 patients went through the operation successfully. The time from injury to operation ranged from 5 to 21 days (8.7 ± 2.6 days). The operation time ranged from 35 to 150 min (75 ± 29 min). The intraoperative blood loss was from 250 to 1400 ml (440 ± 153 ml). According to the Matta score, the fracture reduction was evaluated as excellent in 131 cases, good in 31 cases, and poor in 16 cases, with an overall excellent and good rate of 91%. Four patients suffered wound fat liquefaction and healed after fresh dressing. All patients were followed up for 1 to 5 years without wound infection. All fractures were healed. At the last follow‐up, the modified Merle d'Aubigne‐Postel score results were evaluated as excellent in 125 cases, good in 26 cases, and fair in 27 cases, with an overall excellent and good rate of 84.8%. Postoperative complications included six cases of traumatic arthritis of the hips and two cases of femoral head necrosis.

**Conclusion:**

The LRA with an AIAP can help expose, reduce, and fix anterior and posterior columns as well as the quadrilateral area of the acetabulum, which is capable of improving the reduction quality of complex acetabular fractures and shortening surgical time and blood loss, thus reaching a good clinical efficacy.

## Graphical abstract

This study designed an acetabular integrative anatomical plate (AIAP) according to the anatomical morphology of the acetabulum in 1000 Chinese people, and explored its application in the fresh complex acetabular fractures through the lateral‐rectus approach (LRA), which achieved the accurate reduction and rigid fixation of complex acetabular fractures during operation, decreased the operation time and intraoperative bleeding, thus reaching a satisfactory operation effect.

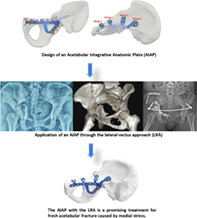

## Introduction

Complex acetabular fractures are mostly high energy injuries. Due to the special anatomical site of the acetabulum, irregular anatomical morphology and various fracture types, acetabular fracture is relatively difficult to be reduced and fixed, and is one of the most challenging fractures faced by orthopedic surgeons, and its treatment outcome is often not satisfactory.^1^, ^2^

The purpose of acetabular fracture surgery is to restore the matching relationship between the femoral head and acetabulum, reconstruct and maintain concentric reduction of the hip joint, so as to reduce postoperative pain, the incidence of traumatic arthritis and the risk of internal fixation failure.^3^ The anterior force types of complex acetabular fractures mainly include double column acetabular fracture, anterior column and posterior semi‐transverse acetabular fracture, and T‐type acetabular fracture. The common surgical approaches include the ilioinguinal approach, modified Stoppa approach, the lateral‐rectus approach and the pararectus approach. Among them, the lateral‐rectus approach has been widely used in treating complex acetabular fractures because of its minimal trauma, full exposure of anterior and posterior columns and the quadrilateral area of the acetabulum.^4^, ^5^

At present, reconstruction plates combined with column screws have been mainly used for acetabular fracture fixation. But due to the irregular anatomical shape of the acetabulum, reconstruction plates that need to be bent during operation have poor fit, leading to the increase of the operation time and intraoperative bleeding. In addition, the combined fixation of multiple plates also has some shortcomings such as mutual interference between plates, which affects the excellent and good rate of reduction of acetabular fractures. Nowadays, 3D printing has been widely used in the treatment of complex acetabular fractures.^6^ Because the design of acetabular integrative anatomical plates (AIAPs) accorded with the anatomical morphology of the acetabulum in 1000 Chinese people, our AIAPs can achieve the accurate reduction and rigid fixation of complex acetabular fractures during operation,^7^, ^8^ which can significantly contribute to the excellent and good rate of fracture reduction, the operation time, intraoperative bleeding, and operation effect.

**Fig. 1:**
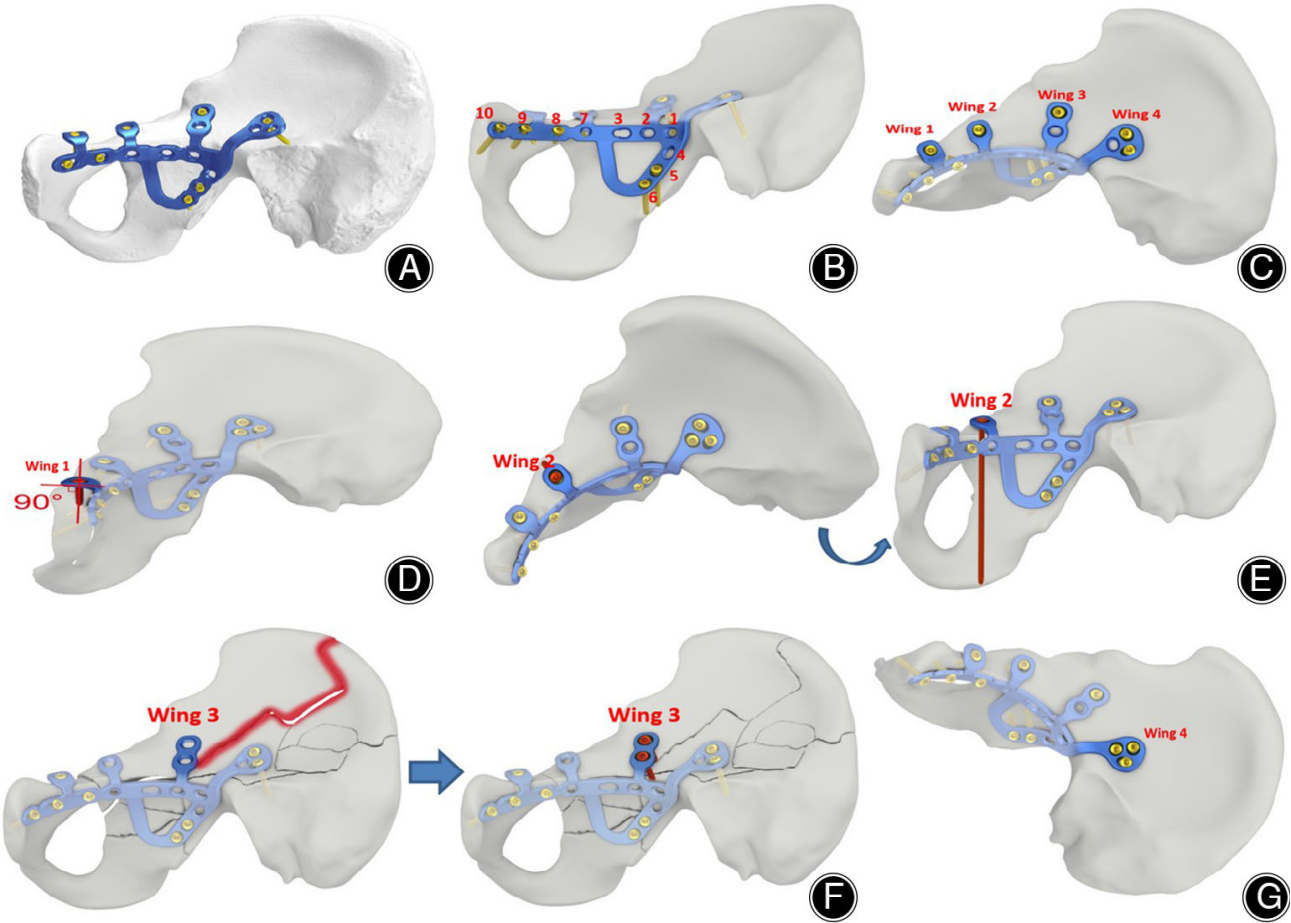
Diagram of the acetabular integrative anatomical plate model. (A) Streamline design of the AIAP; (B) The main body of the AIAP; (C) Four wings separately extend from the greater sciatic foramen to the pubic tubercle; (D) Wing 1 is located at the outer upper part of the pubic tubercle, and the placement direction of its screw is from the outer top to the inner bottom pointing to the lower branch of the pubis, cross fixed with the anterior column plate screws; (E) Wing 2 is above the anterior wall of the acetabulum near the obturator ring, and its screw can point outward and downward to fix the fracture block of the anterior wall of the acetabulum, or can point to the lower edge of the acetabulum that forms a triangular stability with the main body of the plate; (F) Wing 3 points to the anterior inferior iliac spine, and its proximal hole points to the posterior wall of the acetabulum for reducing the displacement of the acetabulum, while its distal hole points to the outer plate of the ilium for correcting the external rotation deformity of the iliac wing; (G) Wing 4 with three oval screw holes in different directions is above the greater sciatic foramen, which is the toughest part of the acetabulum.

The AIAP (Patent number CN217472056U, CN209032596U) is designed on the basis of the normal pelvic anatomical data, which is a streamline design with double columns, four wings and multi‐planes (Figure 1A). The main body of the plate consists of an acetabular anterior column from the lower part of the greater sciatic foramen along the true pelvic edge to the pubic symphysis, an acetabular posterior column from the lower edge of the greater sciatic foramen to the ischial spine, thus realizing the integrated design of the acetabular anterior and posterior columns. It is divided into six holes to 10 holes to meet the needs of different body shapes (Figure 1B). The screw direction of holes 1 and 2 of the main body of the AIAP is from the inside to the outside. The screw direction of holes 4–6 is 45° oblique downward, which is convenient for screw placement. At the same time, while tightening the screws, the posterior column can also be pressed by the oblique upward pull of these screws. A transverse bar is designed to connect with the anterior column and posterior column at the lower edge of the acetabulum to form the triangular stability for the medial side of the acetabulum, which also contributes to the comminuted fracture blocks in the quadrilateral area of the acetabulum. Above the true pelvic margin, four different winged branches separately extend from the greater sciatic foramen to the pubic tubercle (Figure 1C). There is a single‐hole wing (Wing 1) protruding from the outside of the pubic tubercle, and the screw placement direction is almost 90° fixed to the pubic symphysis with thole 9 of the main body plate, which can effectively prevent the screw from loosening and prolapse (Figure 1D). Another single‐hole wing (Wing 2) is above the anterior wall of the acetabulum near the obturator ring, and its screw can point outward and downward to fix the fracture block of the anterior wall of the acetabulum, or can point to the lower edge of the acetabulum that forms a triangular stability with the main body of the plate (Figure 1E). A 2‐hole wing (Wing 3) points to the anterior inferior iliac spine, and its proximal hole points to the posterior wall of the acetabulum for reducing the displacement of the acetabulum, while its distal hole points to the outer plate of the ilium for correcting the external rotation deformity of the iliac wing (Figure 1F). The toughest part of the acetabulum is above the greater sciatic foramen, where has a wing (Wing 4) with three oval screw holes in different directions to ensure that the plate can be fixed on the strongest bone of the acetabulum (Figure 1G). The whole structure of the plate is designed according to the anatomical morphology of the anterior column, posterior column, anterior wall, the quadrilateral area, superior pubic branch and iliac fossa bone surface of the acetabulum, which finally forms an integrated acetabular anatomical plate with two columns, four wings and multi‐planes across the great ischial foramen, ischial spine and pubic symphysis.

The AIAP is characterized by multi‐plane auxiliary reduction and fixation, which is suitable for complex acetabular fractures under stress on the inside of the acetabulum, especially for complex acetabular fractures with obvious displacement, complex comminution, difficult fixation with common reconstruction plate, as well as pelvic osteoporotic fractures in the elderly. The operation can be exposed by the lateral‐rectus approach (LRA) and implanted with the plate to complete the anatomical reduction and to provide strong internal fixation for the acetabulum.

The purposes of this study were to: (i) design and improve the AIAP; (ii) explore the surgical method of LRA combined with an AIAP for complex acetabular fractures; and (iii) evaluate the clinical efficacy of AIAPs for complex acetabular fractures.

## Materials and Methods

### Patients

This study has been approved by the Ethics Committee of our hospital (Ethics Review of No. 201508006). Informed consent was obtained from all included subjects.

From March 2016 to September 2021, a total of 178 cases of acetabular fractures was treated with the AIAP *via* the LRA. According to Judet–Letournel classification,^9^ there were 66 cases of anterior column with posterior semi‐transverse fracture, 104 cases of double‐column fracture, eight cases of T‐type fracture. In this cohort, 42 patients were combined with pelvic ring injury, 37 with limbs fractures, and 27 with injury of bladder, rectum and urethra. The above statistics, as well as mechanism of injury, combined injury are shown in Table 1.

**TABLE 1:** Patient demographic and injury data

Parameter	Value	Percentage
Age (years)	57.8 ±16.7 (18–93)	
Sex		
Male	138	77.5
Female	40	22.5
Mechanism of injury		
Traffic accident	124	69.7
Fall from height (greater than standing)	42	23.6
Fall (from standing height)	12	6.7
Fracture classification		
Anterior column with posterior semi‐transverse fracture	66	37.1
Double‐column fracture	104	58.4
T‐type fracture	8	4.5
Combined injury		
Pelvic ring fracture	42	23.6
Limb fracture	37	20.8
Bladder, rectal and/or urethral injuries	27	15.2
Hypertension, coronary heart disease, diabetes and other medical diseases	43	24.2

### Inclusion and Exclusion Criteria

Inclusion criteria included: (i) fresh acetabular fractures (less than 3 weeks old); (ii) acetabular fractures involving anterior and posterior columns and quadrilateral area; (iii) treated with the AIAP *via* the LRA; and (iv) not less than a one‐year follow‐up period.

Exclusion criteria included: (i) other internal fixation methods; (ii) injured for more than 3 weeks; (iii) posterior approach for posterior wall fractures, or combined anterior and posterior approach due to other reasons; and (iv) serious underlying diseases or unstable vital signs that cannot tolerate operation and anesthesia.

### Perioperative Management

After admission, routine preoperative examinations were performed for all patients. The supracondylar traction of the affected femur was performed. Oral Rivaroxaban (10 mg, qd) was given to patients to prevent deep venous thrombosis. For patients with deep venous thrombosis, Rivaroxaban (15 mg, bid) was given for 21 days and then Rivaroxaban (20 mg, qd) was given to maintain treatment. Symptomatic treatment was performed for patients with combined injuries and medical diseases. And surgical treatment was performed after the patient's condition was stable. The color Doppler ultrasonography of lower extremities was performed 1 day before operation to rule out deep venous thrombosis of the lower extremities.

**Fig. 2:**
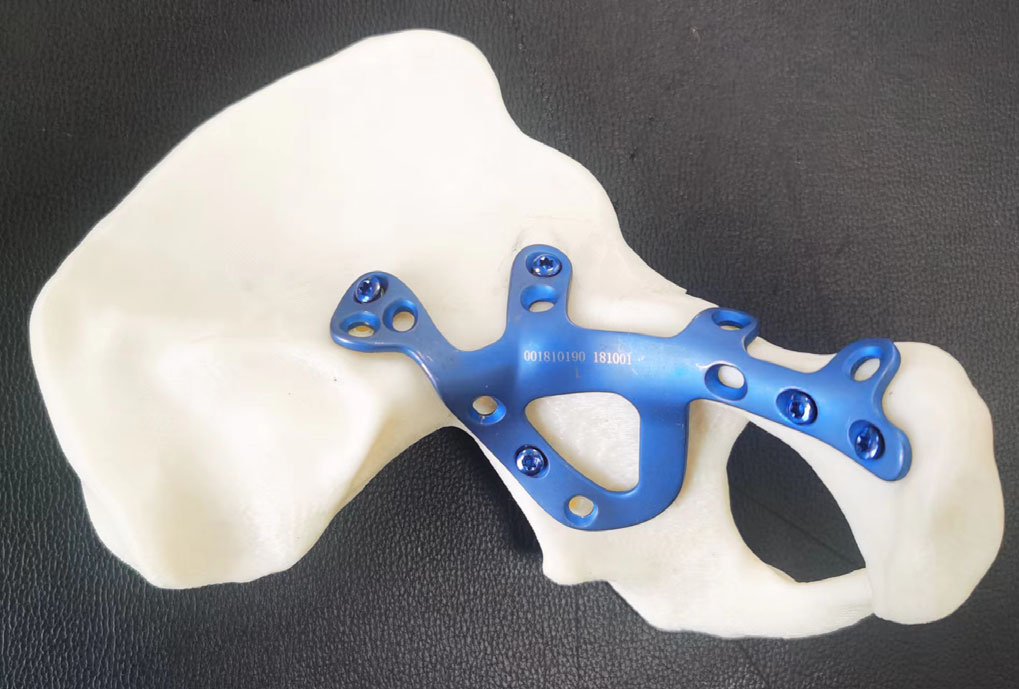
Verify the matching of the AIAP on the half pelvic mirror model.

All patients were examined with a standard pelvic X‐ray and 64‐slice CT scan (Toshiba 64 slice, Tokyo, Japan). If patients' economic condition was acceptable, their 3D fracture models of 1:1 were printed to understand the fracture type and displacement. The mirror model of the healthy side of the pelvis was printed by using the mirror image principle. The AIAP was matched with the mirror model (Figure 2). The operation was simulated on the model to determine the position, direction and length of the inserted screws. The plate and screws were disinfected for operation preparation.

### Design of the AIAP

The AIAP was designed based on the anatomical morphology of the acetabulum in 1000 Chinese people. First, pelvic CT data were collected in the DICOM (Digital Imaging and Communications in Medicine) format. Continuous images with a layer thickness of 1 mm were scanned and recorded on a CD in the DICOM format. Then the DICOM files were imported into Mimics 19.0 software (Materialise Software, Leuven, Belgium) to generate a 3D model of the pelvis. The 3D mirrored model of the healthy hemipelvis was generated for the AIAP design. After the STL file of the 3D mirrored hemipelvic model was imported into Geomagic Studio 2012 (3D systems, Rock Hill, SC, USA), the contour of the AIAP on the mirrored pelvis was designed and extracted by using Geomagic Studio software. Subsequently, the models of the AIAP and the mirrored pelvis were imported into Solidworks professional 2015 software (Dassault Systèmes Solidworks Corp, Waltham, MA, USA) for creation of the insertion path of screws on the AIAP. Finally, the design of the AIAP was completed and the file was saved as an STL file for 3D post production.

### Surgical Procedure

After tracheal intubation general anesthesia, patients were placed in the supine position. All fractures were exposed by the LRA.^10^ After exposing the pubic symphysis to the whole medial side of the acetabulum and the fracture end below the sacroiliac joint through the medial window and the middle window of the LRA, lower limb traction was assisted for reduction. The anterior column, anterior wall, posterior column and the quadrilateral area were reduced in proper order, temporarily fixed with Kirschner wire to maintain reduction. After general reduction of the fracture, the AIAP was placed into the medial window through the middle window. The edge of the posterior column of the plate was close to the inferior edge of the sciatic foramen. The anterior Wing 1 was located on the outside of the pubic tubercle. The plate was pressed by the top rod to fit with the acetabular bone surface as far as possible. The front of the plate was clamped close to the pubic branch with bone holding forceps. The screws were inserted through holes of Wings 1 and 4 to fix the plate on the acetabulum. Then the reduction forceps were used to clamp the plate and the outer plate of the ilium to further reduce and compress the fracture with the help of the plate. The iliac wing fracture with obvious displacement could be reduced through the lateral window of the LRA, and fixed with ordinary reconstruction plate or screw. After the fracture reduction was satisfactory and the plate was well attached to the bone surface, the acetabulum was fixed by anterior and posterior, internal and external compression with screws according to the characteristics of screws. Intraoperative C‐arm fluoroscopy was used to observe the fracture reduction and the position of the plate and screws.

### Postoperative Management

After the operation, patients maintained a semi‐recumbent position with hip flexion of both lower limbs to relax external iliac vessels and prevent deep venous thrombosis of lower extremities. Fluid diet was allowed after anal exhaust. Drainage was removed when the fluid volume was less than 50 ml/day or within 48 h. Low‐molecular‐weight heparin was used routinely 6 h postoperatively assisted with pressure trousers of both lower limbs to prevent deep venous thrombosis. After patients' condition was stable, Pelvic X‐ray and CT scan were performed to evaluate the reduction of the fracture. Isometric contraction training of lower limb muscles could be carried out 24 h after operation. Patients were permitted toe‐touch weight‐bearing 6–10 weeks after surgery while full weightbearing depended on the patient's general condition and fracture healing state.

### Postoperative Assessment and Follow‐up

Postoperative pelvic X‐ray and CT scan were performed at 4 weeks, 12 weeks, 6 months and 1 year after operation to examine the reduction and healing of fractures. According to Matta's criteria,^11^ the acetabular reduction quality was assessed. The results are divided as follows: fracture displacement < 1 mm is excellent, 1–3 mm is good, >3 mm is poor. The modified Merle d'Aubigne‐Postel scoring system^12^ was used to evaluate hip joint function during one‐year follow‐up after operation: excellent (18 points), good (15–17 points), fair (13–14 points), and poor (<13 points).

### Statistical Analysis

The data were analyzed by SPSS 25.0 (SPSS Inc., Chicago, IL, USA). Categorical data were reported as numbers and percentages. And continuous data were presented as mean with standard deviation (SD), including the time from injury to operation, the operation time and the intraoperative bleeding.

## Results

### Demographic Results

All 178 patients were treated with AIAPs *via* the LRA. Among them, there were 138 males and 40 females, with an average age of 57.8 ± 16.7 years old. The time from injury to operation was 5–21 days (mean 8.7 ± 2.6 days).

### Intraoperative Results

**Fig. 3:**
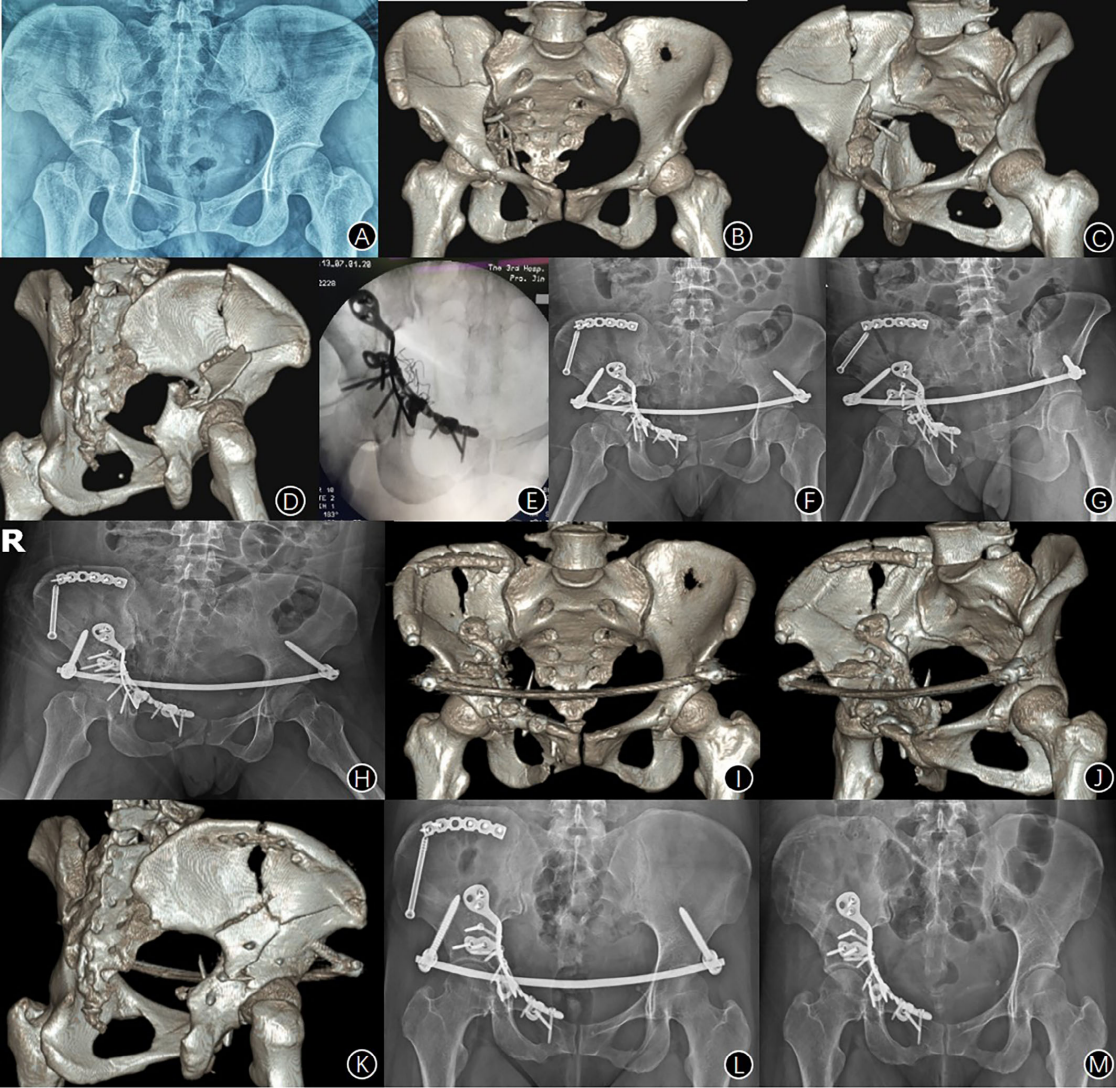
A 36‐year‐old female with fresh acetabular both‐column fracture, who had been admitted to the hospital 2 h after the traffic injury, was treated with an AIAP via the LRA. (A) Preoperative radiograph of anteroposterior view; (B–D) Preoperative three‐dimensional reconstructions; (E) Intraoperative radiograph of anteroposterior view; (F–H) Postoperative radiograph of anteroposterior, iliac oblique and obturator oblique views; (I‐K) Postoperative three‐dimensional reconstructions; (L) Anteroposterior radiograph at 3 months after operation; (M) Anteroposterior radiograph at 1 year after operation.

The operation time was 35–150 min (mean 75 ± 29 min). The intraoperative bleeding was 250–1400 ml (mean 440 ± 153 ml). During the operation, X‐ray films showed that the AIAP was attached to the bone surface with well reduction, and the screw did not enter the acetabular fossa (Figure 3). The number of effective fluoroscopy during the operation was 3–10 times, with an average of 5.5 times.

### Postoperative Clinical Functional Results

According to Matta's criteria, the results were excellent in 131cases, good in 31 cases and poor in 16 cases, with an overall excellent and good rate of 91%. At the last follow‐up, the hip function was scored according to the modified Merle d'Aubigne‐Postel score system: excellent in 125 cases, good in 26 cases, fair in 27 cases, with a total excellent and good rate of 84.8%.

### Imaging Results

Postoperative pelvic X‐ray and CT scan showed good reduction.

### Complications

**Fig. 4:**
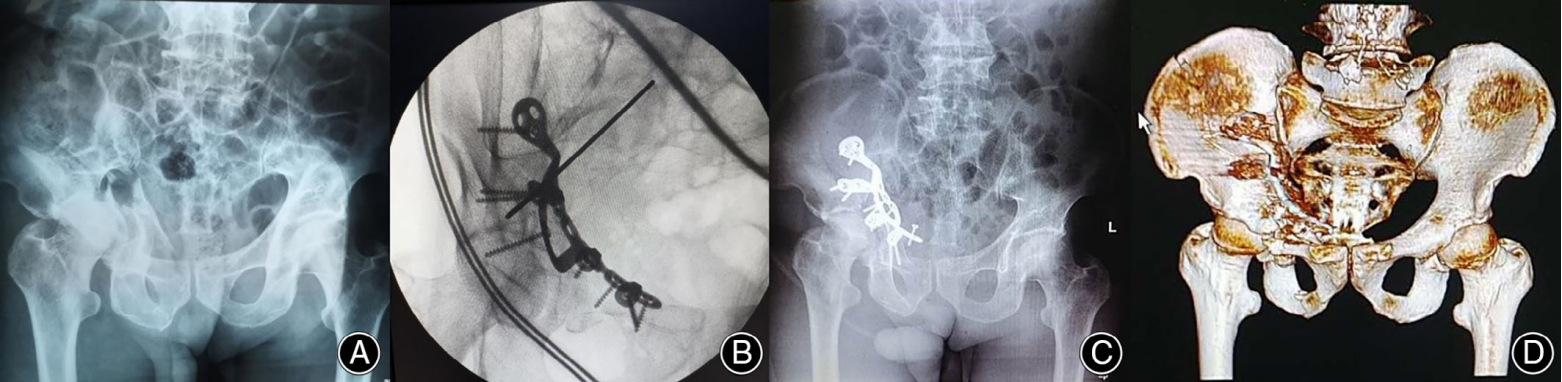
A male with both column fracture. (A) Preoperative radiograph of anteroposterior view; (B) Intraoperative radiograph of obturator oblique view; (C, D) A radiograph of anteroposterior and three‐dimensional reconstruction showed obvious screw loosening during the follow‐up after surgery.

Wound fat liquefaction occurred in four cases and healed after dressing change. No deep infection occurred. Deep venous thrombosis of lower extremities occurred in 13 cases within 1 week after operation, and disappeared by thrombolysis and other symptomatic treatment. Fifteen cases had symptoms such as medial thigh sensory hypoesthesia and adduction weakness of hip joint, which recovered within 3 months without special treatment. There were no complications such as lateral femoral cutaneous nerve injury and sciatic nerve injury. During the follow‐up of 1 to 5 years, all fractures healed. Screw loosening at pubic tubercle occurred in two cases without re‐displacement (Figure 4). No plate fracture occurred. Six cases had traumatic hip arthritis and two cases had osteonecrosis of femoral head after surgery. A typical case is shown in Figure 3.

## Discussion

The acetabulum is an important structure for mechanical conduction and normal activity in the human body, with a deep position and complex surrounding structures. It is difficult to reduce and fix the acetabular fractures with conventional plates, especially when they involve the quadrilateral plate disruption. Thus, it is necessary to design a fully fitted and personalized plate for patients with acetabular fractures. In the current study, the LRA^5^ provides good exposure of both columns and the quadrilateral area of the acetabulum. And our study suggested that the AIAP *via* the LRA was a minimally invasive surgical approach for the treatment of complex acetabular fractures by improving the quality of fracture reduction, achieving satisfactory fixation, reducing surgical time and surgical trauma.

### Characteristics of the AIAP

The AIAP, which has a streamlined anatomical shape with both‐column and four wings, is designed and improved with the following characteristics: (i) the curvature shape of the plate is designed based on the surface shape of the acetabular CT reconstruction model of healthy adults to ensure its perfect fit. (ii) The integrative design is based on the mechanical transmission of the acetabulum and the distribution characteristics of the bone trabecula, extending from the sacroiliac joint to the anterior column (pubic symphysis) and the posterior column (ischial spine), to achieve the integrative fixation of the anterior and posterior columns. (iii) The triangular frame of the quadrilateral area contributes to the reduction and stability of the quadrilateral area fracture. (iv) Each of the four wings has its own functions. The screw of Wing 1 is almost 90 degrees to the screws on the inner side of the plate, which can avoid the loosening of the screws here. There were only two cases of screw loosening in this group after surgery. The screw of Wing 2 can fix the anterior wall of the acetabulum, or can be inserted to form triangular stability of the acetabulum together with the column plate. Wing 3 can effectively correct the rotation of the iliac wing. Wing 4 locates on the strongest part of the acetabulum, ensuring that the force is transmitted to the anterior or posterior column. (v) The funnel‐shaped oval design of the screw holes enables screws to be placed at an angle of about 30° in each direction, facilitating the screws into the strongest bone site and avoiding the screws from entering the hip joint and fracture line. There was no screw entering the acetabular fossa in our study.

### Advantages of the AIAP in Clinical Application

The AIAP has unique advantages in the reduction and fixation of complex acetabular fractures caused by inner force, which mainly display in the following aspects. (i) As the integrative streamline design of the plate conforms to the direction of human mechanics transmission, the function of hip joint and lower limbs will not be affected after fracture reduction and healing. (ii) The anatomical shape of the plate can be pressed by clamping for assisting the further anatomical reduction of the fracture and reducing the time of bending the plate during the operation. The excellent and good rate of fracture reduction in this study was 91%. (iii) The integrative design of the plate connecting with anterior column, posterior column, the quadrilateral area and the iliac wing can avoid the mutual interference in the placement positions of multiple plates. (iv) The screws near the pubic tubercle can form an angle of 90°, which reduce the unstable fixation and screw loosening rate here. It is especially suitable for elderly patients combined with osteoporosis or those combined with pubic fractures close to the pubic symphysis, avoiding the trauma caused by the fixation of the plate across the pubic symphysis. (v) As each screw hole is designed far away from the the acetabular nail placement danger zone, the screw will be basically prevented from entering the acetabular fossa and will not affect hip arthroplasty in the future when the plate is positioned appropriately during the operation. And it only needs to verify the reduction quality and the position of the plate and screws by fluoroscopy after reduction and fixation under direct vision, which can reduce the times of intraoperative fluoroscopy. In our study, there were only two patients who had internal fixation looseness during follow‐up, but no fracture re‐displacement occurred. The excellent rate of fracture reduction was 91%, much higher than that of the traditional fixations (78.9%–81.0%).^13^, ^14^ The incidence of traumatic arthritis was only 3.4% (6/178) in our study, which was much lower than that of the traditional fixations (29.6%–30.7%).^15^, ^16^ These results could indicate that the design of the AIAP is efficient and its clinical application can have significant effects on reduction and fixation of complex acetabular fractures.

### Feasibility of Using the AIAP via the LRA for Acetabular Fractures

The whole internal side of the acetabulum can be directly viewed after the external peritoneal exposure, the interactive exposure through the medial window and the middle window of the LRA.^17^ The exposure mode of LRA is different from the ilioinguinal approach (outward‐inside exposure) and Stoppa approach (outward‐inside exposure), which is convenient for the AIAP placement and multi‐directional screw placement. The following key points should be noted in the operation. (i) Take supine operation under general anesthesia, disinfect the affected lower limbs and bandage for lower limb traction during operation. For young and middle‐aged patients, sufficient muscle relaxation and controlled blood pressure reduction can be maintained during operation, so as to facilitate intraoperative exposure and reduce bleeding. (ii) Do not separate external iliac vascular bundles, inferior epigastric vessels, spermatic cord (round ligament of uterus) between the medial window and the middle window, which should be stretched as a whole for protecting iliac vessels from traction injury. (iii) After the fracture end is exposed under periosteum, lower limb traction and Schanz nail of anterior superior iliac spine can be used to assist reduction. The anterior column should be restored, followed by the reduction of posterior column and quadrilateral area. After the acetabular contour is generally restored, Kirschner wire can be used for temporary fixation. (iv) The AIAP should be inserted below the iliac vascular bundle through the middle window to the medial window (noting that the plate should not press the obturator nerve). Wing 1 should be located on the pubic tubercle. The rear column edge of the plate should be attached to the lower edge of the sciatic foramen. Press the plate against the bone surface with a top rod. Screws of Wing 1 and Wing 4 should be inserted for maintaining the position of the plate. Then the plate and the lateral wall of iliac bone should be clamped with pelvic reduction forceps for further reduction. After the anatomical reduction and fixation under direct view, the reduction and the position of the plate and screws can be verified by fluoroscopy.

The LRA can fully expose the front of the sacroiliac joint. The bleeding of presacral venous plexus can be handled *via* the LRA under direct vision, which can significantly reduce the amount of intraoperative bleeding and the risks of operation. The AIAP can satisfy further reduction of the fracture. It also improves the strength of fixation by overcoming the disadvantage of uncertain stability of multiple plate fixation. The complete anatomical design of the plate avoids the reduction loss caused by the non‐attachment of the plate, and saves the time of pre‐bending the plate during the operation.

Compared with previous literature, the surgical methods in our study have advantages in terms of surgical time, blood loss, and excellent reduction rate, without significant differences in functional satisfaction. Sagi *et al*.^18^ treated 57 cases of acetabular fractures with reconstruction plates and column screws through the modified Stoppa approach. The average surgical time was 263 min. The average blood loss was 750 ml. The excellent reduction rate was 92%. And the functional satisfaction rate was 90%. Märdian *et al*.^19^ retrospectively compared the average surgical time of the ilioinguinal approach and the pararectus approach, which was 256 min and 233 min, respectively. The excellent and good reduction rates of the above two approaches were 73.3% and 100%. And their satisfactory hip joint function rates were 96.6% and 100%, respectively. Zha *et al*.^20^ used a new internal fixation device to treat acetabular fractures, with an average surgical time of 110.3 ± 30.8 min, intraoperative bleeding volume of 950.6 ± 348.6 ml, excellent reduction rate of 83.4%, and functional satisfaction rate of 87.5%. The fixation device could be adjusted to compress the quadrilateral area, but its volume was relatively large. However, among the 178 cases in our study treated by the LRA with the AIAP, the average operation time (75 ± 29 min) and the average intraoperative blood loss (440 ± 153 ml) were significantly less than those of traditional operations.^21^, ^22^, ^23^ The LRA with the AIAP for treatment of the acetabular fractures can greatly reduce surgical trauma, complications, hospitalization costs and rehabilitation time.

### Limitations

Compared to common acetabular reconstruction plates, the AIAP has the following shortcomings. (i) Due to large volume of the plate, it is difficult to insert the plate if the exposure is insufficient and the placement skills of the plate are not mastered. (ii) As the plate is a multi‐plane design with wide screw insertion angle, it can be more easily inserted by the LRA or the pararectus approach than by other approaches. (iii) As the LRA is intrapelvic exposure, it is difficult to insert the screws of the posterior column plate and the screws of the anterior column plate in obese patients. (iv) It is difficult or even impossible to take out the plate after surgery because of tissue adhesion in the pelvic cavity, so it is necessary to communicate clearly with patients before operation.

Apart from the shortcomings mentioned above, this study still had other limitations including lack of control experiment, relatively short follow‐up period and small sample. Thus, a larger number of patients should be observed and a longer follow‐up incorporated for further study.

### Conclusion

The AIAP combined with the LRA is a promising treatment for fresh acetabular fracture caused by medial stress, which has great advantages in reducing operation time and intraoperative bleeding, minimizing surgical trauma, improving quality of reduction and fixation.

## Authorship Declaration

All authors listed meet the authorship criteria according to the latest guidelines of the International Committee of Medical Journal Editors, and all authors are in agreement with the manuscript.

## Funding Information

This study was supported by the National Natural Science Foundation of China (82072411), National Key Research and Development Program of China (2022YFC2504303), Innovation fund cultivation project of National Clinical Research Center for Orthopedics Sports Medicine & Rehabilitation (2021‐NCRC‐CXJJ‐PY‐06), the Special Program of Guangdong Frontier and Key Technological Innovation (No. 2015B010125006).

## Conflict of Interest Statement

All authors listed declared that there are no conflicts of interest to this work. There are no commercial or associative interest that represents a conflict of interest in connection with the work submitted. We declare that we have no conflicts of interest.

## Author Contributions

Shicai Fan: Conceptualization, Methodology, Formal analysis, Investigation, Writing—Original Draft, Writing—Review & Editing. Qiguang Mai: Data Curation, Visualization. Tao Li: Data Curation, Investigation. Hua Wang: Data Curation, Investigation. Cheng Yang: Formal analysis, Investigation. Huang Hai: Formal analysis, Investigation. Jianwen Liao: Formal analysis, Investigation. Yingze Zhang: Writing—Review & Editing.

## Supporting information


**Video S1.** Reduction and fixation of acetabular fractures with an AIAP.
